# Incidence, Patterns, and Characteristics of Patients With Acute Kidney Injury Requiring Renal Replacement Therapy in Cancer Settings

**DOI:** 10.7759/cureus.48627

**Published:** 2023-11-10

**Authors:** Summra Siddiq, Zakia Hussain, Janmohamed Mubarakali, Anam Nazir

**Affiliations:** 1 Department of Nephrology, King’s College Hospital, London, GBR; 2 Department of Gynecology and Obstetrics, Royal Stoke University Hospital, Stoke-on-Trent, GBR; 3 Department of Nephrology, New Cross Hospital, Wolverhampton, GBR; 4 Department of Internal Medicine, Pakistan Kidney and Liver Institute and Research Center, Lahore, PAK

**Keywords:** renal replacement therapy (rrt), diabetes mellitus, kidney disease improving global outcomes (kdigo) classification, intensive care unit, acute kidney injury(aki))

## Abstract

Background

Cancer patients are at increased risk of multi-organ failure due to either the primary disease burden or certain non-cancer-related risk factors. Among the most common complications is acute kidney injury (AKI), which is frequently seen in cancer settings. Among patients with cancer, the incidence of renal injury reaches up to 12.5%. However, critical care units have a much higher incidence, up to 50%. This study aimed to describe the characteristics of Asian populations with AKI with a background of malignancy, along with risk factors and outcomes.

Materials and methods

A retrospective tertiary-care single-center study was conducted in the intensive care unit (ICU). It included 182 cancer patients with AKI who were followed over a 36-month period.

Results

Our results revealed a mortality rate of 50.5% among cancer patients with AKI, with the highest mortality rate being among those with solid and hematologic malignancies. Common predisposing factors were sepsis (28%), dehydration (18.1%), and hypotension (9.9%). Several drugs were found to be among the most toxic agents, including vancomycin, colistin, nonsteroidal anti-inflammatory drugs, angiotensin receptor blockers, amphotericin, and certain chemotherapeutic drugs (especially cisplatin). A strong association was found between the length of ICU stay and mortality (p=<0.05), indicating a reduced survival rate with prolonged hospital stay even in critical care settings.

Conclusion

In summary, AKI in cancer patients increases their mortality due to a variety of risk factors. However, the early involvement of onconephrology and a multidisciplinary approach will result in better outcomes.

## Introduction

Kidney Disease Improving Global Outcomes (KDIGO) defines acute kidney injury (AKI) as “an absolute increase in serum creatinine (SCr) of at least 0.3 mg/dl (26.5 mmol/L) within 48 hours or by a 50% increase in SCr from baseline within seven days, or a urine volume of less than 0.5 mL/kg/h for at least six hours” [1-3]. Cancer patients with impaired renal function have a lower survival rate compared with the general population due to underlying disease burden, an immunocompromised state, micronutrient deficiency, volume depletion, electrolyte imbalances, and a neutropenic state, which makes such patients susceptible to multi-organ damage [1-4].

AKI in cancer patients can worsen the outcomes and diminish the adequacy of cancer treatments [1-3]. Several new therapies have been developed for various malignancies; however, they are associated with several adverse effects, including AKI [4-6]. Furthermore, managing cancer patients with AKI with chemotherapeutic agents that contain nephrotoxic elements poses a significant challenge [7-9]. 

Every year, the global incidence of cancer increases, as does the incidence of AKI in the cancer population, which ranges from 12% to 66.5% [1]. Multiple causes have been identified as triggering factors that lead to renal damage, especially low-volume states, poor nutrition, and repeated episodes of infection against a background of immunosuppression.

Depending on their clinical condition, different patients receive different types of renal replacement therapies (RRTs). Although there is a paucity of evidence surrounding any particular modality of RRT with favorable outcomes in cancer patients, the initiation of dialysis is considered to be one of the worst prognostic factors. Several studies have demonstrated a poor prognosis in cancer patients when they require RRT [8-11].

Because of geographical variations in the types of cancers, clinical presentations, associated co-morbidities, and socioeconomic factors, sufficient data is lacking to guide the treatment and prevention of renal injury among cancer patients in Pakistan. To establish such data, a retrospective observational study was conducted on the Pakistani population. This study highlighted the basic characteristics of cancer patients, including the underlying etiological factors, mortality rates, and associated long-term complications with repeated episodes of renal injury.

## Materials and methods

A retrospective single-center study was conducted among cancer patients at Shaukat Khanum Memorial Cancer Hospital and Research Center( SKMC & RC), Pakistan. The Institutional Review Board (IRB) of Shaukat Khanum Memorial Cancer Hospital and Research Center (SKMC & RC) approved this study. All clinical investigations were conducted in accordance with the principles of the Declaration of Helsinki. Furthermore, all procedures were followed in accordance with the current local and national ethical standards of the IRB and Scientific Review Committee.

**Figure 1:**
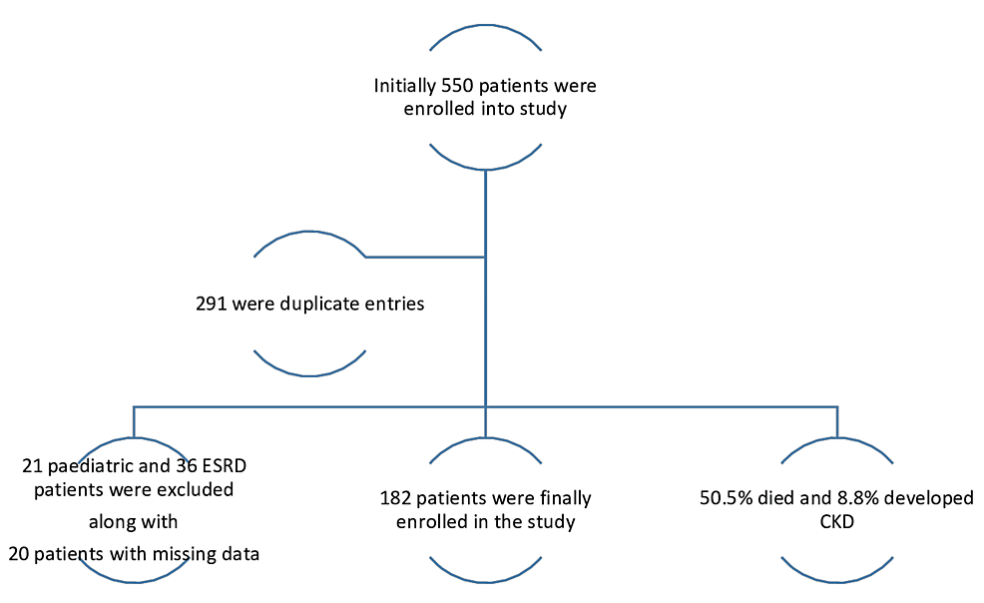
Flowchart showing the distribution of the study population

The objective of this study was to determine the incidence, patterns, and characteristics of cancer patients with AKI. Additionally, this study aimed to determine the mortality rate among different cancer types and its association with the length of stay in the intensive care unit (ICU). All patients with AKI admitted to the ICU between January 2016 and December 2018 were included in the study. Using a non-probability consecutive sampling technique, 550 cancer patients were initially enrolled, among whom 290 were found to be duplicate entries. Figure 1 presents a flowchart of the study population's distribution.

Of the remaining patients, 21 who belong to the pediatric age group and 36 who have end-stage renal disease were excluded. All patients who were >18 years old and had AKI at presentation on top of underlying chronic kidney disease (CKD) were included in the study (Table 1). A total of 182 patients were enrolled and followed over a three-year period. Table 1 indicates the number of patients with AKI and CKD.

**Table 1:** Number of patients with acute kidney injury and underlying chronic kidney disease CKD: chronic kidney disease

CKD-Stage	No. of patients (n)	Percentage (%)
Stage- I	2	1.1
Stage- II	14	7.7
Stage-III	14	7.7
Stage-IV	11	6.0
None	141	77.5

The baseline demographics of the patients were reviewed using the hospital information system (HIS), including their age, gender, co-morbidities, type of cancer, medications, chemotherapeutic agents, and etiology of renal injury. Then, their laboratory results were reviewed, including albumin, potassium, bicarbonate, creatinine, serum urea nitrogen, and hemoglobin levels. Serum creatinine was measured using a simple blood test with the gold standard isotope dilution mass spectrometry method. CKD was defined based on the National Kidney Foundation’s Kidney Dialysis Outcomes Quality Initiative, and the estimated glomerular filtration rate (eGFR) was calculated using the Modification of Diet in Renal Disease equation.

Next, data analysis was conducted using IBM SPSS Statistics for Windows, Version 22.0 (released 2013; IBM Corp., Armonk, New York, United States). Continuous data were summarized as mean and standard deviation, while categorical data were summarized as frequency and percentage. A significant correlation between risk factors was assessed using the chi-squared test; p <0.05 was considered statistically significant. The data were graphically presented using bar charts, graphs, and tables. Table 2 presents an overview of the baseline characteristics of the cancer patients enrolled in this retrospective study.

**Table 2:** Baseline characteristics of the enrolled cancer patients

Baseline characteristics	No. of patients/Percentage (%)
Median age (range), years	47 (18- 76)
Gender: Male	124 (68.1)
Female	58 (31.9)
Underlying malignancy: Prostate	16 (8.8)
Bladder	15 (8.2)
Colon	22 (12.1)
Breast	14 (7.7)
Pancreas	11 (6.0)
Hematologic	37 (20.2)
Germ cell tumors	20 (11.0)
Others	47 (26.0)
Hypertension	63 (34.6)
Diabetes mellitus	43 (23.1)
Pre-existing chronic kidney disease	38 (20.9)
Single functioning kidney	18 (9.9)
Ischemic heart disease	9 (4.9)
Others	12(6.6)
Hemoglobin, g/dL	10 ±2.6
Albumin, g/L	2.9 ±0.89
Urine output, mL/min	44 ±38.4
Baseline creatinine, mg/dL	1.35 ±1.0
Creatinine at admission, mg/dL	5.5 ±3.9
Systolic BP (range), mmHg	80- 165
Diastolic BP (range), mmHg	40 -70

## Results

**Figure 2:**
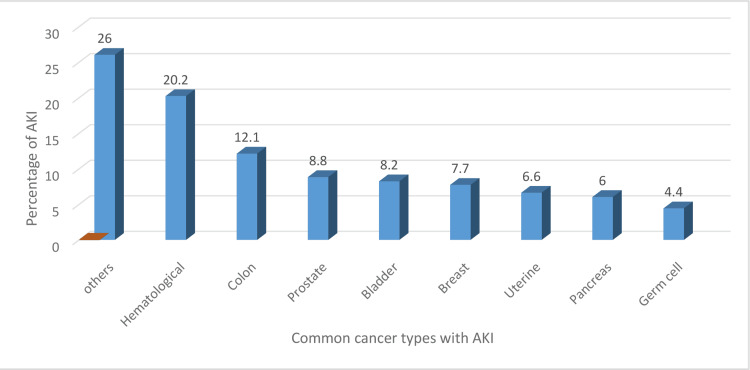
Percentage of acute kidney injury in different cancer populations

This study’s findings revealed that out of a total of 182 cancer patients with AKI, 68.1% (n=124) were men and 31.9% (n=58) were women. Their median age was 47 years (Table 1). Common malignancies with an increased risk of AKI included hematologic malignancies (20.0%), breast cancer (7.7%), colon cancer (12.1%), and lower urinary tract cancer involving the prostate and bladder (16% and 15%, respectively) (Table 1). The bar chart in Figure 2 presents the percentage of AKI in these different cancer populations.

Furthermore, several comorbidities were identified as risk factors for AKI, including hypertension (34.6%; (n=63)), diabetes mellitus (23.1%; (n=43)), pre-existing CKD (20.9%; (n=38)), and a single functioning kidney (9.9%; (n=18)).

Moreover, this study observed that most of the cancer patients had low hemoglobin (10 ± 2.6), low albumin, and decreased urine output; however, none of these factors proved significant in determining the prognosis of acute renal damage (Table 1). The peak creatinine observed among these patients at admission was Cr 5.5 ± 3.9 mg/Dl, with the pre-renal variety of AKI at 63.7% (n=116), followed by intra- and post-renal types at 29.1% (n=53) and 7.1% (n=13), respectively (Table 1).

**Figure 3:**
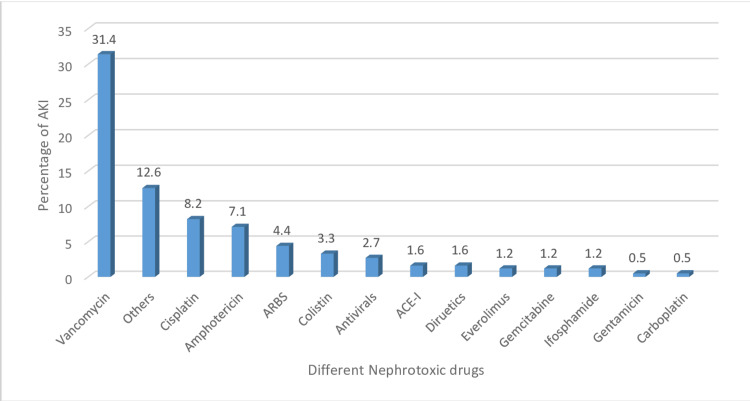
Different nephrotoxic drugs that contribute to acute kidney injury by percentage

Several antibiotics, anti-virals, anti-fungal, and chemotherapeutic agents were identified as being involved in causing renal damage through direct toxicity and tubular injury. Among these agents, vancomycin contributed 21.4% (n=39), amphotericin 7.1% (n=13), colistin 3.3% (n=6), acyclovir 2.7% (n=5), and chemotherapeutic agents-cisplatin 8.2% (n=15), everolimus 1.2%, and gemcitabine 1.2%. Certain other drugs were also responsible for adding insult to renal injury, including angiotensin-receptor blockers (ARBs) (4.4%) and non-steroidal anti-inflammatory drugs (NSAIDs) (4.4%). Figure 3 presents a bar chart of the different nephrotoxic drugs that contribute to acute kidney injury.

Apart from drugs and other co-morbidities, a number of different cancer- and non-cancer-related risk factors were identified as causative agents of renal damage. These risk factors are presented in Table 3.

**Table 3:** Risk factors for acute kidney injury among cancer patients ACE-I: angiotensin-converting enzyme inhibitors; ARBs: angiotensin receptor blockers; NSAIDs: non-steroidal anti-inflammatory drugs

Non-cancer-related risk factors	Cancer-related risk factors
1. Age >60 years	1. Infections secondary to immunocompromised state
2. Hypertension	2. Hematologic malignancies
3. Diabetes mellitus	3. Urinary tract obstruction
4. Pre-existing chronic kidney disease	4. Tumor lysis syndrome
5. Single functioning kidney	5. Anti-cancer treatment
6. Drugs (vancomycin, colistin, ACE-I, ARBs, and NSAIDs)	6. Agents (cisplatin, everolimus, efosfamide, and gemcitabine)

The study results demonstrated that patients with an average age above 60 years with a background history of hypertension, a single-functioning kidney, or diabetes mellitus are associated with increased risk. Among non-cancer-related risk factors, an immunocompromised state, repeated infections, tumor lysis syndrome, and hematologic malignancies increase risk (Table 3).

In addition, the most common etiological factors among cancer patients with AKI were found to include sepsis (28%; (n=51)), dehydration (18.1%;(n=33)), hypotension (9.9%; (n=18)), obstructive uropathy (14.3% (n=26)), presence of a solitary kidney (7.2%;(n=13)), tumor lysis syndrome(2.8%; (n=5)), and others (18.6% (n=34)). Table 4 lists the most common etiological factors.

**Table 4:** Common etiological factors of acute kidney injury among cancer patients MAHA: micro-angiopathic hemolytic anemia; TLS: tumor lysis syndrome

Etiological factors	No. of patients/Percentage (%)
Hypotension	18 (9.9)
Dehydration	33 (18.1)
Sepsis	51 (28)
Tumor lysis syndrome	5 (2.8)
Obstructive uropathy	26 (14.3)
Single functioning kidneys	13 (7.2)
Disease burden	2 (1.1)
Others	34 (18.6)
MAHA	1 (0.5)
Arrhythmias	6 (3.3)
Drugs	27 (14.8)

Based on the KDIGO classification, the majority of patients had stage III AKI (82.4%; (n=150)). Table 5 presents the number of patients by the type and stage of AKI.

**Table 5:** Types of acute kidney injury and stages among cancer patients AKI: acute kidney injury; KDIGO: Kidney Disease Improving Global Outcomes

KDIGO stage of AKI	No. of patients (%)	Type of AKI	No. of patients (%)
Stage I	5.0 (2.7)	Pre-renal	116 (63.7)
Stage II	26 (14.3)	Renal	53 (29.1)
Stage III	151 (83)	Post-renal	13 (7.2)

**Figure 4:**
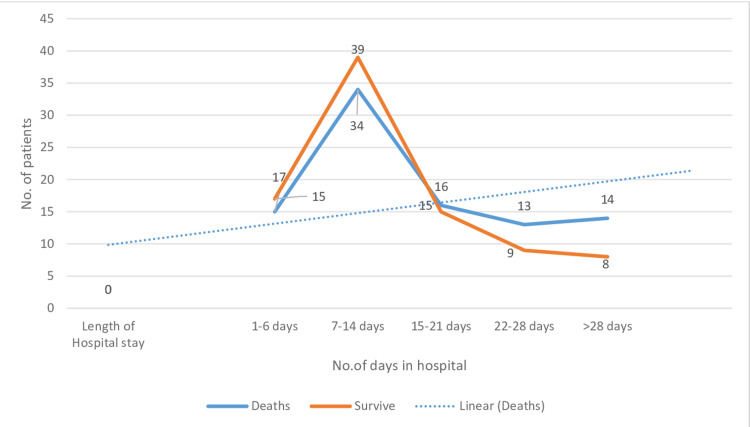
Graph indicating a reduced survival rate among patients with increasing length of hospital stay

The average length of hospital stay observed among critically ill patients was 13.8 ± 9.2 days, and the average stay in the ICU was 6.8 ± 9.1 days (Table 6), indicating the existence of a significant correlation with mortality (p=0.01). The prolonged length of ICU stay is proportionally related to increased mortality and a reduced survival rate (Figure 4).

**Table 6:** Primary and secondary outcomes of acute kidney injury in an oncology setting LAMA: leave against medical advice; AKI: acute kidney injury

Outcomes	No. of patients/ Percentages (%)	p-value
Primary outcomes	-	-
Deaths	92 (50.5%)	-
Survival	88 (48.5%)	-
LAMA	1 (0.5%)	-
Palliative care referral	1 (0.5%)	-
Secondary outcomes	-	-
Length of ICU stay (days)	6.8 ± 9.1	0.01
Total length of hospital stay	13.8 ± 9.7	0.44
Readmission within 30 days	35 (19)	-
AKI on re-admission	18 (9.9)	-
Development of chronic kidney disease	16 (8.8)	0.03

The mortality rate increased with a prolonged ICU stay, as indicated by the dotted lines. This suggests a linear relationship between prolonged ICU stays and a higher mortality rate.

Moreover, this study found the highest mortality rate among patients affected by sepsis (37.0%), followed by nephrotoxic drugs (19.6%), dehydration (15.2%), and hypotension (10.9%). Table 7 presents the mortality rates of cancer patients with different AKI risk factors.

**Table 7:** Mortality rates for cancer patients with various risk factors for acute kidney injury TLS: tumor lysis syndrome

Risk factors for acute kidney injury	Associated deaths (No. of patients =92)	Mortality rate percentage (50.5%)
Dehydration	14	15.2
Nephrotoxins	18	19.6
Sepsis	34	37.0
Hypotension	10	10.9
Arrhythmias	4	4.3
Single functioning kidney	4	4.3
TLS	4	4.3
Disease burden	2	2.2
Obstructive uropathy	2	2.2

Furthermore, certain cancer types were found to be at an increased risk of AKI-related mortality, namely hematologic cancer (mortality rate of 25.0%) and the following solid tumors: breast cancer (10.9%), colon cancer (19.6%), prostate cancer (15.2%), and lung cancer (4.5%). Table 8 presents the cancer types with the highest mortality rates.

**Table 8:** List of cancers with the highest mortality rates

Cancer type	Men	Women	Total no. of deaths	Mortality rate (%)
Hematologic	15	8	23	25
Breast	1	9	10	10.9
Genitourinary: Male	14	0	14	15.2
Genitourinary: Female	0	4	4	4.3
Lung	5	0	5	5.4
Gastrointestinal	13	5	18	19.6
Others	11	7	18	19.6
Total	59	33	92	50.5

The primary outcome of this study was a mortality rate of 50.5% among cancer patients with AKI, and furthermore, 48.5% of cancer patients survived. A significant association was found to exist between the length of hospital stay and mortality in the ICU (p <0.05). The secondary outcome was the development of CKD in a significant number of patients (8.8%, p<0.03), with repeated episodes of AKI. Table 6 presents the primary and secondary outcomes of AKI in an oncology setting.

## Discussion

AKI is frequently observed in critically ill patients [2-4], especially in cancer settings [5-7]. This is due to multiple risk factors associated with an immunocompromised state [7-9]. The KDIGO classification of AKI used in this study includes serum creatinine (SCr) and urine output as criteria for assessing renal injury [1]. Based on previous studies, it is well known that the risk of AKI is significantly higher in cancer patients compared with the non-cancer population [9,10,12]. Similar results were obtained in this study, indicating that cancer patients are prone to AKI. A higher incidence of cancer was found among men compared with women at 68.1% and 31.9%, respectively, which is likely due to unequal access to medical facilities between the two genders. It is also influenced by the increased frequency of genitourinary malignancies in men (Table 2), mainly prostate and bladder cancer, which were found to have incidence rates of 8.8% and 8.2%, respectively (Figure 2). Similar results have been obtained in other studies [10,11], in addition to low socioeconomic status being a critical factor in this regard.

Furthermore, hematologic malignancies remain among the most common cancers that cause renal damage [11,13,14]. The chemotherapeutic agents used for their treatment are associated with an increased risk of kidney damage (Figure 3) [15,16,17], with cisplatin having the highest toxicity of 8.2%. Volume status assessment and adequate kidney perfusion are necessary to avoid such toxic effects, along with dose reduction according to renal clearance. However, multiple other pre-existing comorbidities, both cancer and non-cancer-related, also contribute to AKI in addition to primary disease, including hypertension (34.6%), diabetes (23.1%), congenital solitary functioning kidney (9.9%), and underlying CKD (20.9%) (Table 3).

Most of the patients in this study suffered from stage III AKI secondary to mainly pre-renal etiological factors. Common gastrointestinal symptoms associated with malignancy and anti-cancer medications include nausea, vomiting, diarrhea, and poor oral intake, all of which contribute to severe dehydration, hypotension, and reduced renal perfusion (Table 5). Certain parameters were observed in this study, such as hypotension, decreased urine output, dry mucous membranes, and postural drops indicating volume depletion, that have also been reported in other studies [10,11]. Early intervention with fluid resuscitation and optimal nutrition can help to prevent AKI.

Furthermore, this study showed that sepsis is the most common etiological factor that causes AKI, as seen in 51% of cases (Table 4). Immunocompromised states and compromised nutritional status in cancer patients make them prone to bacterial, viral, and fungal infections, which can lead to multi-organ failure [12]. Previous studies have demonstrated that certain antibiotics and antifungal medications used to treat infections are also responsible for additional nephrotoxicity [7,18,19]. Vancomycin-associated renal damage was observed in 21.4% of our study population, followed by amphotericin-associated damage in 7.1%, and then colistin, which caused a 3.3% increased risk of AKI (Figure 3). These findings highlight that although these cancer patients are at an increased risk of infection due to their immunocompromised state, the prophylactic or therapeutic use of these drugs significantly increases the risk of further renal damage [9,17,18,20,21].

In such situations, it is crucial to weigh the benefits against the risks of administering such medication. Several other authors have reported similar results, such as Salahudeen [10], who reported a strong association between the use of antibiotics and AKI, although this finding was not statistically significant. Furthermore, Lahoti [12] reported a strong and independent association between AKI and antibiotics, and another study also had similar findings [19]. In the present study, we observed that a significant number of patients were affected by antibiotic-associated kidney injury (32.3%); however, we were unable to establish any statistically significant correlation (p=0.39).

Drug toxicity can be reduced by selecting the least toxic agent with low doses [21-23], which will assist in improving renal outcomes. Cisplatin, a commonly used anti-cancer chemotherapeutic agent for solid tumors, was associated with nephrotoxicity in 8.2% of cases (Figure 3) in our study population, which is similar to results reported in other studies [20-22]. However, these drugs are the mainstay of treatment for these cancer patients. With reduced renal clearance, the chances of toxicity increase; therefore, the early involvement of onconephrology [23] can help to overcome kidney injury through early intervention and a multidisciplinary approach. Similarly, the damaging effects of non-cancer-related risk factors could be reduced through patient education, including the optimal control of hypertension, and diabetes, dietary modifications, and the maintenance of adequate hydration and nutrition with regular follow-up.

Different RRT modalities were used to salvage kidneys, but none of them proved to be useful. Depending on the specific indication (Table 9) and hemodynamic state, patients were offered hemodialysis, continuous RRT, or isolated ultrafiltration.

**Table 9:** Indications for renal replacement therapy in critically ill cancer patients in the intensive care unit

Reason for dialysis initiation	No. of patients/Percentage %
Stage III acute kidney injury	126 (69.0)
Severe metabolic acidosis	17 (9.3)
Refractory hyperkalemia	10 (5.5)
Tumor lysis syndrome	8.0 (4.4)
Uremic encephalopathy	8.0 (4.4)
Hypercalcemia	7.0 (3.8)
Pulmonary edema	6.0 (3.3)

Different etiological factors lead to AKI that requires renal replacement therapy (RRT), with stage III AKI being the most common reason for dialysis. However, different risk factors have different mortality rates, and infections are among the most common reasons for a higher mortality rate (37%) (Table 6), followed by drug-induced toxicity (19.6%) and hypovolemia (15.2%), respectively. Differences also exist between different malignancies, with hematologic diseases noted to have the highest mortality rate of 20.2% (Table 8). Although these cancers are common in young patients with better physiological reserves, the risk of recurrent hospital admission makes increased mortality and morbidity likely. An increasing length of hospital stay, including in the ICU, was found to be directly proportional to a higher mortality rate, as indicated in Figure 4. ICU stays longer than two weeks were associated with reduced survival. Furthermore, the number of deaths in the ICU increased even after the provision of all medical facilities, including RRT, among cancer populations [24,25]. Moreover, a significant correlation existed between the length of ICU stay and mortality (p<0.01). Different modalities of renal replacement have been used, but none of them proved superior or effective at prolonging survival [26,27]. The primary outcome of this study was a mortality rate of 50.5% among cancer patients with AKI (Table 6).

Additionally, a significant number of patients were readmitted within 30 days of discharge with another episode of renal injury (19%), which made them prone to developing progressive renal disease and de novo CKD. Out of 18 (9.9%) patients who had frequent episodes of AKI, the data indicated that 16 (8.8%) developed CKD, which is another statistically significant finding (p=0.03). Multiple risk factors are involved in the development of CKD, especially repeated episodes of acute kidney injury [28,29]. To improve outcomes for cancer patients, it is essential to involve a multidisciplinary approach at an earlier stage. This is evidenced by the fact that, even in critical care settings with RRT and intensive care available, the mortality rate remained high when compared with the general population with AKI.

This study has some limitations. First, it was a retrospective study based on medical records; thus, data recording errors cannot be excluded. To avoid such errors, a prospective case-control study would have been ideal in this scenario. Secondly, multicenter data should be collected and compared to establish the existence of typical characteristics among the Pakistani cancer population suffering from renal injury for a more generalized impact.

## Conclusions

In summary, AKI is common in cancer settings, and multiple risk factors trigger a deterioration of renal function, although some of them are modifiable. The high mortality rate seen in critically ill patients RRT reaches 50.5%. With the development of onconephrology, certain risks are preventable through employing early intervention, using renally adjusted doses of chemotherapeutic agents, avoiding febrile illnesses, and maintaining adequate volume status. Undoubtedly, following a multidisciplinary approach would result in a better prognosis. However, further studies are required to prove these assumptions.

## References

[1] Roy AK, Mc Gorrian C, Treacy C, Kavanaugh E, Brennan A, Mahon NG, Murray PT (2013). A comparison of traditional and novel definitions (RIFLE, AKIN, and KDIGO) of acute kidney injury for the prediction of outcomes in acute decompensated heart failure. Cardiorenal Med.

[2] Lam AQ, Humphreys BD (2012). Onco-nephrology: AKI in the cancer patient. Clin J Am Soc Nephrol.

[3] Campbell GA, Hu D, Okusa MD (2014). Acute kidney injury in the cancer patient. Adv Chronic Kidney Dis.

[4] Gallieni M, Cosmai L, Porta C (10.1159/000484970). Acute Kidney Injury in Cancer Patients. Contrib Nephrol.

[5] Rosner MH, Perazella MA (2019). Acute kidney injury in the patient with cancer. Kidney Res Clin Pract.

[6] Kitchlu A, McArthur E, Amir E (2019). Acute kidney injury in patients receiving systemic treatment for cancer: a population-based cohort study. J Natl Cancer Inst.

[7] Safdar A, Ma J, Saliba F (2010). Drug-induced nephrotoxicity caused by amphotericin B lipid complex and liposomal amphotericin B: a review and meta-analysis. Medicine (Baltimore).

[8] Lameire N, Vanholder R, Van Biesen W, Benoit D (2016). Acute kidney injury in critically ill cancer patients: an update. Crit Care.

[9] Farooqi S, Dickhout JG (2016). Major comorbid disease processes associated with increased incidence of acute kidney injury. World J Nephrol.

[10] Salahudeen AK, Doshi SM, Pawar T, Nowshad G, Lahoti A, Shah P (2013). Incidence rate, clinical correlates, and outcomes of AKI in patients admitted to a comprehensive cancer center. Clin J Am Soc Nephrol.

[11] Cardwell CR, O'Sullivan JM, Jain S, Hicks BM, Devine PA, McMenamin ÚC (2021). Hormone therapy use and the risk of acute kidney injury in patients with prostate cancer: a population-based cohort study. Prostate Cancer Prostatic Dis.

[12] Lahoti A, Kantarjian H, Salahudeen AK (2010). Predictors and outcome of acute kidney injury in patients with acute myelogenous leukemia or high-risk myelodysplastic syndrome. Cancer.

[13] Klastersky J, Ameye L, Maertens J (2007). Bacteraemia in febrile neutropenic cancer patients. Int J Antimicrob Agents.

[14] Canet E, Zafrani L, Lambert J (2013). Acute kidney injury in patients with newly diagnosed high-grade hematological malignancies: impact on remission and survival. PLoS One.

[15] Perazella MA, Moeckel GW (2010). Nephrotoxicity from chemotherapeutic agents: clinical manifestations, pathobiology, and prevention/therapy. Semin Nephrol.

[16] Malyszko J, Kozlowska K, Kozlowski L, Malyszko J (2017). Nephrotoxicity of anticancer treatment. Nephrol Dial Transplant.

[17] Lameire N, Kruse V, Rottey S (2011). Nephrotoxicity of anticancer drugs-an underestimated problem?. Acta Clin Belg.

[18] Elyasi S, Khalili H, Dashti-Khavidaki S, Mohammadpour A (2012). Vancomycin-induced nephrotoxicity: mechanism, incidence, risk factors and special populations. A literature review. Eur J Clin Pharmacol.

[19] Feld R (1999). Vancomycin as part of initial empirical antibiotic therapy for febrile neutropenia in patients with cancer: pros and cons. Clin Infect Dis.

[20] Launay-Vacher V, Rey JB, Isnard-Bagnis C, Deray G, Daouphars M (2008). Prevention of cisplatin nephrotoxicity: state of the art and recommendations from the European Society of Clinical Pharmacy special interest group on cancer care. Cancer Chemother Pharmacol.

[21] Horie S, Oya M, Nangaku M (2018). Guidelines for treatment of renal injury during cancer chemotherapy 2016. Clin Exp Nephrol.

[22] Riad MA (2022). A potentially less nephrotoxicity of carboplatin over cisplatin as radiosensitizer in head-neck cancer. Asian Pacific J ournal of Cancer Biology.

[23] Perazella MA (2012). Onco-nephrology: renal toxicities of chemotherapeutic agents. Clin J Am Soc Nephrol.

[24] Seylanova N, Crichton S, Zhang J, Fisher R, Ostermann M (2020). Acute kidney injury in critically ill cancer patients is associated with mortality: a retrospective analysis. PLoS One.

[25] Ganguli A, Sawinski D, Berns JS (2015). Kidney diseases associated with haematological cancers. Nat Rev Nephrol.

[26] Maccariello E, Valente C, Nogueira L (2011). Outcomes of cancer and non-cancer patients with acute kidney injury and need of renal replacement therapy admitted to general intensive care units. Nephrol Dial Transplant.

[27] Benoit DD, Hoste EA, Depuydt PO (2005). Outcome in critically ill medical patients treated with renal replacement therapy for acute renal failure: comparison between patients with and those without haematological malignancies. Nephrol Dial Transplant.

[28] Ellis MJ, Parikh CR, Inrig JK, Kanbay M, Patel UD (2008). Chronic kidney disease after hematopoietic cell transplantation: a systematic review. Am J Transplant.

[29] Hingorani S (2006). Chronic kidney disease in long-term survivors of hematopoietic cell transplantation: epidemiology, pathogenesis, and treatment. J Am Soc Nephrol.

